# Supplementary material for: An Inducible System for Silencing Establishment Reveals a Stepwise Mechanism in Which Anchoring at the Nuclear Periphery Precedes Heterochromatin Formation
Source: Cells. 2021 Oct 20;10(11):2810. doi: 10.3390/cells10112810 (PMC8616196; doi:10.3390/cells10112810)
Supplement: Supplementary file 1 [file cells-10-02810-s001.zip › cells-1429156-supplementary.pdf]

Supplementary Material

An Inducible System for Silencing Establishment Reveals a Stepwise Mechanism in Which Anchoring at the Nuclear Periphery Precedes Heterochromatin Formation

Isabelle Loïodice [1], Mickael Garnier [1], Ivaylo Nikolov [1] and Angela Taddei [1,2,*]

[1] Nuclear Dynamics unit, CNRS, Institut Curie, PSL University, Sorbonne Université, 75005 Paris, France; isabelle.loiodice@curie.fr (I.L.); mickael.garnier@curie.fr (M.G.); ivaylo.l.nikolov@gmail.com (I.N.)

[2] Cogitamus Laboratory, F-75005 Paris, France

* Correspondence: angela.taddei@curie.fr

Table S1: Yeast strains used in this study

Name	Parent	Genotype	Source
**W303-1 derived strains:**		***MATa ade2-1 can1-100 leu2-3,112 his3-11,15 trp1-1 ura3-1***	
yAT1		*MATa ade2-1 trp1-1 his3-11 his3-15 ura3-1 leu2-3 leu2-112 can1-100 rad5-*	(1)
yAT126	yAT1	*MATa ade2-1::ADE2*	(2)
yAT2000	yAT2001	*MATa lys2::EADE2I-lacO+(TRP1) nup49::mCherry-NUP49(URA3) his3::Hisp-GFP-LacI(HIS3)*	This study
yAT2001		*MATa lys2::EADE2I-lacO+(TRP1) nup49::mCherry-NUP49(URA3)*	This study
yAT2059	yAT2001	*MATa lys2::EADE2I-lacO+(TRP1) nup49::mCherry-NUP49(URA3) leu2:: Hisp-GFP-LacIR(LEU2)*	This study
yAT2078	yAT2059	*MATa lys2::EADE2I-lacO+(TRP1) nup49::mCherry-NUP49(URA3) leu2:: pGalS(NAT)-GFP-LacIR(LEU2)*	This study
yAT2156	yAT2000	*MATa lys2::EADE2I-lacO+(TRP1) nup49::mCherry-NUP49(URA3) his3::Hisp-GFP-LacI(HIS3) sir3Δ::KanMX4 hmlΔ::ClonNath*	This study
yAT2370	yAT2078	*MATa lys2::EADE2I-lacO+(TRP1) nup49::mCherry-NUP49(URA3) leu2:: pGalS(NAT)-GFP-LacIR(LEU2) hmlΔ::KanMX sir3Δ::HPH*	This study
yAT3259	yAT2078	*MATa lys2::EADE2I-lacO+(TRP1) nup49::mCherry-NUP49(URA3) leu2:: pGalS(NAT)-GFP-LacIR(LEU2) sir4Δ::HPH HMLΔ::KanMX*	This study
yAT3258	yAT2078	*MATa lys2::EADE2I-lacO+(TRP1); nup49::mCherry-NUP49(URA3); leu2:: pGalS(NAT)-GFP-LacIR(LEU2) hmlΔ::HPH sir1Δ::KanMX*	This study
yAT3420		*MATa lys2:: lacO+(TRP1) Nup49::mCherry-NUP49(URA3) leu2::Hisp-GFP-LacIR(LEU2)*	This study
yAT3471	yAT3420	*MATa lys2:: lacO+(TRP1) Nup49::mCherry-NUP49(URA3) leu2::pGalS(NAT)-GFP-LacIR(LEU2)*	This study
yAT2314	yAT2078	*MATa lys2::EADE2I-lacO+(TRP1) nup49::mCherry-NUP49 (URA3) leu2:: pGalS(NAT)-GFP-LacIR (LEU2) tel1Δ::KanMX*	This study
yAT2904	yAT2078	*MATa lys2::EADE2I-lacO+(TRP1) nup49::mCherry-NUP49(URA3) leu2:: pGalS-(NAT)-LacIR-GFP(LEU2) mec1Δ::His5 sml1Δ::KanMX*	This study

Y01177		*MATa trp1-1 ura3-1 his3-11,15 leu2-3,112 ade2-1 can1-100 hta1 S129A hta2 S129A*	(3)
yAT2965	Y01177	*MATa lys2::EADE2I-lacO+(TRP1) nup49::mCherry-NUP49(URA3) leu2:: pGalS-(NAT)-LacIR-GFP(LEU2) hta1-S129A hta2-S129A*	This study
yAT2995	yAT2965	*MATa lys2::EADE2I-lacO+(TRP1) nup49::mCherry-NUP49(URA3) leu2:: pGalS-(NAT)-LacIR-GFP(LEU2) sir3Δ::KanMX hmlΔ::HPH hta1-S129A hta2-S129A*	This study
**S288C derived strains:** ***MATa ura3-52 leu2Δ1 trp1Δ63 his3Δ200 GAL2***			
yAT1372	FY1679	*MATa/MATα ura3-52/ura3-52; trp1Δ 63/TRP1; leu2Δ 1/LEU2; his3Δ 200/HIS3; GAL2/GAL2*	(4)
yAT1743	yAT1372	*MATa; ura3-52; leu2Δ 1; trp1Δ 63; his3Δ 200; GAL2*	This study
yAT1798	yAT1743	*MATa ade2::His3p-LacI-GFP (LEU2) lys2::LacO+(TRP1)*	This study
yAT1909	yAT1743	*MATa ade2::pGalS(NAT)-GFP-LacIR(LEU2) lys2::LacO+ (TRP)*	This study
yAT2142	yAT1909	*MATa ade2::pGalS(NAT)-GFP-LacIR(LEU2) lys2::LacO+ (TRP) mre11🐦 ::KanMX*	This study

lacO+: lacO repeats provided by David Sherratt (Lau et al. 2003); lacO array of 120 repeats integrated using pAT229 described in (Dubarry et al, 2011).

(1) Thomas and Rothstein. ***Cell. 1989 Feb24;56(4):619-30.*** doi: 10.1016/0092-8674(89)90584-9

(2) Ruault et al.**J Cell Biol. 2011 Feb 7;192(3):417-31.** doi: 10.1083/jcb.201008007.

(3) Masumoto *et al*. ***Nature. 2005 Jul14;436(7048):294-8.*** doi: 10.1038/nature03714

(4) Winston et al. **Yeast Jan 11(1):53-5.** doi: 10.1002/yea.320110107.

Table S2: Primers used in this study

**qPCR primers**		**Forward primer 5'-3'**	**Reverse primer 5'-3'**
oAT588/oAT589	*ACT1*	GGTGGTTCTATCTTGGCTTC	ATGGACCACTTTCGTCGTAT
oAT643/oAT644	*OGG1*	CAATGGTGTAGGCCCCAAAG	ACGATGCCATCCATGTGAAGT
oAT543/oAT544	lacO+ (P1)*	ACCCCGCCTCGTTTGC	GGATCAATTTGAAGAGTAGCTCTAGCA
oAT950/oAT951	lacO+ (P2)*	CCCTTCCTGCTAGAGTTACTTCTTCA	AGCTCTAGCATGGAGAAACGAATT
oAT641/oAT642	LYS2 (P3)	TCGCAAAAATGCCGACAAT	GCTTGTCAAATCTTGGGACCAT
oAT1540/oAT1541	LYS2 (P4)	TGATTTACCATTGGGCACAATTT	AATTTCCGCGGCAAAGG
oAT158/oAT159	ADE2 (P5)#	CGTATGATTGTTGAGGCAGCA	GGCAGGAGAATTTTCAGCATCT
oAT1562/oAT1563	LYS2 (P6)	CGTCAGGGCCAAGGATGA	AGTACCATAGGTGATACCTGCCTTTT
oAT615/oAT616	Tel6R 0,2kb	TGAGGCCATTTCCGTGTGTA	CCCAGTCCTCATTTCCATCAA
oAT637/oAT638	Tel6R 0,5kb	GGCTGGACTACTTTCTGGAATAGC	GAACTGTGCATCCACTCGTTAGG
oAT617/oAT618	Tel6R 1kb	TGATGAATTACAAGGGAACAATGAG	CATCAAACAAGTAGGAATGCGAAA
oAT619/oAT620	Tel6R 2,4kb	TCTCCTTGTCGTCATGTGAAAGTC	AGAGGAGAGTTGCTGCTTCATCA

ADE2 primers were designed to avoid detection of the endogenous ade2-1 alleles (Taddei et al. 2006).
* These primers amplify one specific region of the lacO array found in the pAT229 plasmid.

Cloning primers

To integrate GFP-LacI at ADE2

pAT378 :

oAT884	**SacI site**	GAGA**GAGCTC**CGTTAATGGCTCCTTTTCCA
oAT885	**SacII site**	GAGA**CCGCGG**ATGGCGTTCGTTGTAATGGT
oAT995	**NaeI site**	GAGA**GCCGGC**AGATTTTGGCGTTCCATTTG
oAT996	**SacI site**	GAGA**GAGCTC**AAGAAAGCTCCCCAACCCTA

Site directed mutagenesis on pAT378 to generate a LacI resistant to galactose

oAT1221		ATTAAGTTCTGTCTCGGCGCGTCTGAAGCTGGCTGGCTGGCATAAATATC
oAT1222		GATATTTATGCCAGCCAGCCAGCTTCAGACGCGCCGAGACAGAACTTAAT

Site directed mutagenesis on pAT123 to generate a LacI resistant to galactose

oAT1168		CTGTCTCGGCGCGTCTTAAGCTGGCTGGCTGGCATAA
oAT1169		TTATGCCAGCCAGCCAGCTTAAGACGCGCCGAGACAG

Table S3: Number of experiments for each figure

**Figure 2D**	**RT-QPCR: *ADE2* mRNA**	**Number of experiments**					
	**Time after induction in min**	**0**		**180**	**270**	**360**	**1200**
yAT2059	**wt const.**	3					3
yAT2078	**wt ind.**	5	4	4	3	3	5

**Figure 3A**	**ChIP-QPCR Sir3**	**Number of experiments for each primer**				
	**Primer**	**P1**	**P2**	**P3**	**P4**	**P5**
	**Distance from *E* silencer in kb**	**-3,7**	**-2,2**	**-1**	**-0,5**	**3,2**
yAT2059	**wt const.**	6	8	7	8	7
yAT2078	**wt non-ind.**	17	19	19	18	17
	**wt ind. 45 min**	4	4	4	4	4
	**wt ind. 90 min**	7	7	7	7	7
	**wt ind. 180 min**	12	14	14	13	12
	**wt ind. 270 min**	4	5	5	5	4
	**wt ind. 1200 min**	10	13	12	12	11

**Figure 3B**	**ChIP-QPCR Sir3**	**Number of experiments for each primer**				
	**time in min after induction**	**0**	**45**	**90**	**180**	**270**
yAT2078	**wt ind. at P2**	19	4	7	14	5
	**wt ind. at P4**	18	4	7	13	5
	**wt ind. at P5**	17	4	7	12	4

Figure 3C	ChIP-QPCR H4K16	Number of experiments for each primer				
	time in min after induction	0	45	90	180	1200
yAT2078	wt ind. at P4	8	3	4	4	3
	wt Ind. at P5	7	3	4	3	3

Figure S3A	ChIP-QPCR Sir4	Number of experiments for each primer				
	Primer	P1	P2	P3	P4	P5
	Distance from *E* silencer in kb	-3,7	-2,2	-1	-0,5	3,2
yAT2078	wt non-ind.	5	5	5	5	4
	wt ind. 180 min	4	4	4	4	3
	wt ind. 1200 min	3	4	3	4	3

Figure S3B	ChIP-QPCR Sir3	Number of experiments for each primer				
	Primer	P1	P2	P3	P4	P5
	Distance from *E* silencer in kb	-3,7	-2,2	-1	-0,5	3,2
yAT2078	wt non-ind.	4	4	4	4	4
	wt ind. 20 h	4	4	4	4	4
	wt ind. 48 h	4	4	4	4	4
	wt ind. 72 h	4	4	4	4	4

Figure S3C	ChIP-QPCR H4K16					
	Primer	P1	P2	P3	P4	P5
	Distance from *E* silencer in kb	-3,7	-2,2	-1	-0,5	3,2
yAT2078	wt non-ind.	8	8	8	8	7
	wt ind. 45 min	3	3	3	3	3
	wt ind. 90 min	4	4	4	4	4
	wt ind. 180 min	4	4	4	4	3
	wt ind. 1200 min	3	3	3	3	3

Figure S3D	ChIP-QPCR H4K16/H2A	Number of experiments for each primer				
	time in min after induction	0	45	90	180	1200
yAT2078	wt ind. at P1	8	3	4	4	3
	wt ind. at P2	8	3	4	4	3
	wt ind. at P4	8	3	4	4	3
	wt ind. at P5	7	3	4	3	3

Figure 5A	ChIP-QPCR H2AP/H2A	Number of experiments for each primer				
	Primer	P1	P2	P3	P4	P5
	Distance from *E* silencer in kb	-3,7	-2,2	-1	-0,5	3,2
yAT2059	wt const.	3	3	3	3	3
yAT2078	wt ind. Raf	9	11	10	12	13
	wt ind. 45 min	3	4	3	5	5
	wt ind. 90 min	6	8	7	8	9
	wt ind. 180 min	6	6	7	7	7
	wt ind. 1200 min	4	6	5	8	8

Figure 5B	ChIP-QPCR H2AP/H2A	Number of experiments for each primer			
	Only experiments made the same day for the wt and the mutants are represented				
	**time in min after induction**	**0**	**90**	**180**	**1200**
yAT2078	**wt ind. at P4**	6	3	4	3
yAT2314	***Δtel1* ind. at P4**	6	3	4	3
yAT2904	***Δmec1* ind. at P4**	5	3	3	3

Figure 5C	ChIP-QPCR H2AP/H2A	Number of experiments for each primer				
	**time in min after induction**	**0**	**45**	**90**	**180**	**1200**
yAT2078	**wt ind at P4**	12	5	8	7	8
yAT2370	***Δsir3* ind. at P4**	7	3	3	5	4

Figure 5D	ChIP-QPCR Sir3	Number of experiments for each primer	
	Only experiments made the same day for the wt and the mutant are represented		
	**Time in min after induction**	**0**	**20 h**
yAT2078	**wt ind. at P4**	3	3
yAT2965	***hta-S129A* ind. at P4**	3	3
yAT2078	**wt ind. at P5**	3	3
yAT2965	***hta-S129A* ind. at P5**	3	3

Figure S5A	ChIP-QPCR H2AP	Number of experiments for each primer				
	**Primer**	**P1**	**P2**	**P3**	**P4**	**P5**
	**Distance from *E* silencer in kb**	**-3,7**	**-2,2**	**-1**	**-0,5**	**3,2**
yAT2059	**wt const.**	3	3	3	3	3
yAT2078	**wt non-ind.**	9	11	10	12	13
	**wt ind. 45 min**	3	4	3	5	5
	**wt ind. 90 min**	6	8	7	8	9
	**wt ind. 180 min**	6	6	7	7	7
	**wt ind. 1200 min**	4	6	6	7	8

Figure S5B	ChIP-QPCR H2AP/H2A	Number of experiments for each primer				
	**time in min after induction**	**0**	**45**	**90**	**180**	**1200**
yAT2059	**wt const.at P2**	3	3	3	3	3
	**wt const.at P4**	3	3	3	3	3
yAT2078	**wt ind at P2**	11	4	8	6	6
	**wt ind at P4**	12	5	8	7	8

Figure S5C	ChIP-QPCR H2AP/H2A	Number of experiments for each primer			
	**Distance from End Tel6R**	**0,2 kb**	**0,5 kb**	**1 kb**	**2,4 kb**
yAT2078	**wt**	16	5	5	3
yAT2314	***Δtel1***	11	4	4	4
yAT2904	***Δmec1***	6	3	3	3
yAT2370	***Δsir3***	10	4	4	nd

Figure S5E	ChIP-QPCR H2AP/H2A	Number of experiments for each primer				
	**time in min after induction**	**0**	**45**	**90**	**180**	**1200**
yAT2059	**wt ind at P2**	11	4	8	6	6
	**wt ind at P5**	13	5	9	7	8
yAT2370	***Δsir3* ind at P2**	7	3	3	5	4
	***Δsir3* ind at P5**	6	3	3	4	4

Figure S5F	ChIP-QPCR H2AP/H2A	Number of experiments for each primer			
	**Primer**	**P1**	**P2**	**P3**	**P4**
	**Distance from *E* silencer**	**-3,7**	**-2,2**	**-1**	**-0,5**
yAT1798	**wt const No Silencer**	3	3	3	3
yAT1909	**wt non-ind No Silencer**	4	4	4	4
	**wt ind No Silencer 90 min**	3	3	3	3

	ChIP-QPCR H2A	Number of experiments for each primer			
	**Primer**	**P1**	**P2**	**P3**	**P4**
	**Distance from *E* silencer**	**-3,7**	**-2,2**	**-1**	**-0,5**
yAT1798	**wt const No Silencer**	3	3	3	3
yAT1909	**wt non-ind. No Silencer**	4	4	4	4
	**wt ind No Silencer 90 min**	3	3	3	3

Figure S5G	ChIP-QPCR Sir3	Number of experiments for each primer	
	**Distance from End Tel6R**	**0,2 kb**	**0,5 kb**
yAT2078	**wt**	43	12
yAT2965	***hta-S129A***	11	7
yAT2314	***Δtel1***	9	nd
yAT2904	***Δmec1***	5	nd
yAT2370	***Δsir3***	13	nd

Figure 6A	ChIP-QPCR H2A	Number of experiments for each primer				
	**Primer**	**P1**	**P2**	**P3**	**P4**	**P5**
	**Distance from *E* silencer in kb**	**-3,7**	**-2,2**	**-1**	**-0,5**	**3,2**
yAT2059	**wt const.**	7	7	7	7	6
yAT2078	**wt non-ind.**	11	13	12	13	14
	**wt ind. 45 min**	5	6	5	6	6
	**wt ind. 90 min**	8	10	9	9	10
	**wt ind. 180 min**	7	7	8	8	8
	**wt ind. 1200 min**	6	8	7	9	9

Figure 6B	ChIP-QPCR H2A	Number of experiments for each primer				
	**time in min after induction**	**0**	**45**	**90**	**180**	**1200**
yAT2078	**wt ind at P2**	13	6	10	7	8
	**wt ind at P4**	13	6	9	8	9
yAT2370	***Δsir3* ind at P2**	7	3	3	5	4
	***Δsir3* ind at P4**	7	3	3	5	4

Figure 6C	ChIP-QPCR H2A	Number of experiments for each primer	
	**Primer**	**P1**	**P2**
yAT2000	**wt const.**	6	6
yAT2001	**wt No LacI**	6	6
yAT2156	***Δsir3* const.**	4	4

Figure 6D	ChIP-QPCR H2A	Number of experiments for each primer		
	**Distance from End Tel6R**	**0,2 kb**	**0,5 kb**	**1 kb**
yAT2078	**wt**	26	5	5
yAT2370	***Δsir3***	11	4	4

Figure S6A	ChIP-QPCR H2A	Number of experiments for each primer	
	**Primer**	**P2**	**P4**
yAT2059 H2A	**wt const.**	7	7
yAT2001 H2A	**wt No LacI**	6	6
yAT3420 H2A	**wt const. No Silencer**	3	3

Table S4: Statistics

Kruskal Wallis ANOVA test, *p*-values are corrected using Tukey's range test
$p > 0.01$ is non-significant (ns) and shown in red.

Fig. 2B **Comparison of the intensity of the l*acO E-ADE2-I*/GFP-LacIR spot in G1 between different conditions**

(N; n)<br>**conditions**	(11; 1436)<br>**wt const**	(3; 604)<br>**wt ind. 30min**	(4; 417)<br>**wt ind. 45 min**	(3; 377)<br>**wt ind. 90min**	(5; 725)<br>**wt ind. 180 min**	(14; 2156)<br>**wt ind. 1200 min**
**wt const**		2.068e-08	9.002e-01	2.068e-08	2.068e-08	2.068e-08
**wt ind. 30 min**			2.068e-08			
**wt ind. 45 min**				2.068e-08		
**wt ind. 90 min**					2.068e-08	
**wt ind. 180 min**						1.000e+00

Fig. 2C **Comparison of the localization of the *lacO E-ADE2-I*/GFP-LacIR spot in G1 in between different conditions**

(N; n)<br>**conditions**	(11; 1526)<br>**wt const**	(3; 600)<br>**wt ind. 30 min**	(4; 417)<br>**wt ind. 45 min**	(3; 377)<br>**wt ind. 90 min**	(5; 725)<br>**wt ind. 180 min**	(14; 2213)<br>**wt ind. 1200 min**
**wt const**		3.706e-08	3.706e-08	1.419e-02	1.075e-04	3.205e-03
**wt ind. 30 min**			1.000e+00	8.463e-07		
**wt ind. 45 min**				4.231e-06		
**wt ind. 90 min**					1.000e+00	2.771e-07
**wt ind. 180 min**						3.706e-08
**wt cont. NoSilencer**		5.111e-01	5.787e-01	1.891e-05	6.248e-08	3.706e-08

Fig. S2A **Comparison of the intensity of the *lacO E-ADE2-I*/GFP-LacIR spot in S-G2 between different conditions**

(N; n)<br>**conditions**	(11; 1178)<br>**wt const**	(3; 537)<br>**wt ind. 30 min**	(4; 350)<br>**wt ind. 45 min**	(3; 237)<br>**wt ind. 90 min**	(5; 378)<br>**wt ind. 180 min**	(14; 1566)<br>**wt ind. 1200 min**
**wt const**		2.068e-08	9.987e-01	2.068e-08	2.068e-08	2.068e-08
**wt ind. 30 min**			2.068e-08			
**wt ind. 45 min**				2.068e-08		
**wt ind. 90 min**					6.213e-08	
**wt ind. 180 min**						5.973e-01

Comparison of the intensity of the *lacO E-ADE2-I*/GFP-LacIR spot in between G1 and S-G2 in each condition

**wt const**	1.074e-10
**wt ind. 30 min**	3.812e-01
**wt ind. 45 min**	3.696e-02
**wt ind. 90 min**	3.031e-06
**wt ind. 180 min**	1.060e-10
**wt ind. 1200 min**	1.060e-10

Fig. S2C **Comparison of the localization of the *lacO E-ADE2-I*/GFP-LacIR spot in S-G2 in between different conditions**

(N; n)	(11; 1250)	(3; 540)	(4; 350)	(3; 237)	(5; 378)	(14; 1609)
**conditions**	**wt const**	**wt ind. 30 min**	**wt ind. 45 min**	**wt ind. 90 min**	**wt ind. 180 min**	**wt ind. 1200 min**
**wt const**		3.706e-08	3.706e-08	9.415e-01	2.502e-01	3.437e-07
**wt ind. 30 min**			1.000e+00	3.254e-06		
**wt ind. 45 min**				3.221e-05		
**wt ind. 90 min**					9.914e-01	6.853e-04
**wt ind. 180 min**						6.075e-08
**wt cont. No Silencer**		8.413e-01	9.447e-01	2.162e-05	2.073e-05	3.706e-08

Fig. S2D **Comparison of the localization of the *lacO E-ADE2-I*/GFP-LacIR spot in between G1 and S-G2 in each condition**

**wt const**	5.687e-08
**wt ind. 30 min**	1.122e-03
**wt ind. 45 min**	8.202e-03
**wt ind. 90 min**	2.578e-01
**wt ind. 180 min**	1.694e-02
**wt ind. 1200 min**	8.290e-03

Fig. 4A **Comparison of the localization of the *lacO E-ADE2-I*/GFP-LacIR spot in G1 in between different conditions**

(N; n)	(7; 1017)	(5; 725)	(14; 2213)	(3; 511)	(3; 531)	(8; 1502)
**conditions**	**wt ind. 30_45 min**	**wt ind. 3 h**	**wt ind. 20 h**	***sir3Δ* ind. 30_45 min**	***sir3Δ* ind. 3 h**	***sir3Δ* ind. 20 h**
**wt ind. 30_45 min**				9.648e-01	1.544e-06	2.069e-08
**wt ind. 3 h**					9.079e-01	9.032e-01
**wt ind. 20 h**					2.069e-08	2.068e-08
***sir3Δ* ind. 30_45 min**					2.106e-03	2.109e-08
***sir3Δ* ind. 3 h**						2.858e-01

Fig. 4B **Comparison of the localization of the *lacO E-ADE2-I*/GFP-LacIR spot in G1 in between different conditions**

(N; n)	(14; 2213)	(8; 1502)	(12; 2052)	(9; 1546)
**conditions**	**wt ind.20 h**	***sir3Δ* ind. 20 h**	**wt const. No silencer**	**wt ind.20 h No silencer**
**wt ind. 20 h**			3.768e-09	3.768e-09
***sir3Δ* ind. 20 h**			3.768e-09	7.172e-01
**wt const. No silencer**				3.769e-09

Fig. 4D **Comparison of the intensity of the *lacO E-ADE2-I*/GFP-LacIR spot in G1 between different conditions**

(N; n)	(12; 1933)	(9; 1331)
**conditions**	**wt const. No silencer**	**wt ind. 20 h No silencer**
**wt const. No silencer**		1.060e-10

Fig. S4A **Comparison of the localization of the *lacO E-ADE2-I*/GFP-LacIR spot in G1 in between different conditions**

(N; n)	(14; 2213)	(8;1502)	(5; 991)
**conditions**	**wt ind. 20 h**	***sir3Δ* ind. 20 h**	***sir4Δ* ind. 20 h**
**wt ind. 20 h**		9.561e-10	9.564e-10
***sir3Δ* ind. 20 h**			9.997e-01

Fig. S4B **Comparison of the localization of the *lacO E-ADE2-I*/GFP-LacIR spot in S-G2 in between different conditions**

(N; n)	(7; 890)	(5; 378)	(14; 1609)	(3; 463)	(3; 250)	(8; 965)
**conditions**	**wt ind. 30_45 min**	**wt ind. 3 h**	**wt ind. 20 h**	***sir3Δ* ind. 30_45 min**	***sir3Δ* ind. 3 h**	***sir3Δ* ind. 20 h**
**wt ind. 30_45 min**				6.153e-01	3.013e-04	2.068e-08
**wt ind. 3 h**				1.095e-02	9.999e-01	1.458e-02
**wt ind. 20 h**					2.238e-07	5.064e-04
***sir3Δ* ind. 30_45 min**					7.721e-02	2.068e-08
***sir3Δ* ind. 3 h**						2.646e-02

Fig. 5F **Comparison of the localization of the *lacO E-ADE2-I*/GFP-LacIR spot in G1 in between different conditions**

(N; n)	(5; 725)	(14; 2213)	(3;435)	(4; 708)
**conditions**	**wt ind. 3h**	**wt ind. 20h**	***mec1Δ* ind. 20h**	***hta-S129A* ind. 20h**
**wt ind. 3h**		3.768e-09	3.769e-09	9.606e-09
**wt ind. 20h**			3.468e-01	7.674e-01
***mec1Δ* ind. 20h**				1.489e-01

Fig. S5H **Comparison of the localization of the *lacO E-ADE2-I*/GFP-LacIR spot in G1 in between different conditions**

(N; n)	(8; 1502)	(6; 1017)	(12; 2052)	(9; 1546)
**conditions**	***sir3Δ* ind. 20 h**	***hta-S129A/Sir3Δ* ind. 20 h**	**wt const. No silencer**	**wt ind. 20 h No silencer**
***sir3Δ* ind. 20 h**		2.459e-01	3.768e-09	7.032e-01
***htaS129A/Sir3Δ* ind. 20 h**			2.796e-07	8.034e-01
**wt const. No silencer**				3.770e-09

Fig. 6F **Comparison of the intensity of the *lacO E-ADE2-I*/GFP-LacIR spot in G1 between different conditions**

(N; n)	(4; 541)	(4; 613)
**condition**	**wt const.**	***sir3Δ* const.**
**wt const.**		1.060e-10

Fig. S6B **Comparison of the intensity of the *lacO E-ADE2-I*/GFP-LacIR spot in G1 between different conditions**

(N; n)	(3; 942)	(3; 836)
**condition**	**wt const.**	**wt const. No Silencer**
**wt const.**		1.060e-10

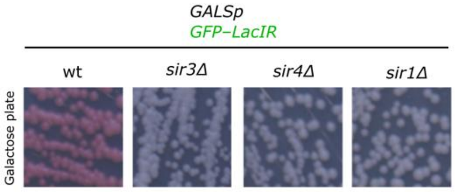

Loïodice et al. Figure S1

Figure S1 related to Figure 1. LacI induced silencing requires Sir3, Sir4 and Sir1. Pictures of yeast colonies bearing the *lys2::lacO E-ADE2-I* locus and expressing the *GALS*p-GFP-LacIR in the WT (yAT2078), in the *sir3Δ* (yAT2370), in the *sir4Δ* (yAT3259), or in the *sir1Δ* (yAT3258) strains streaked on galactose plate.

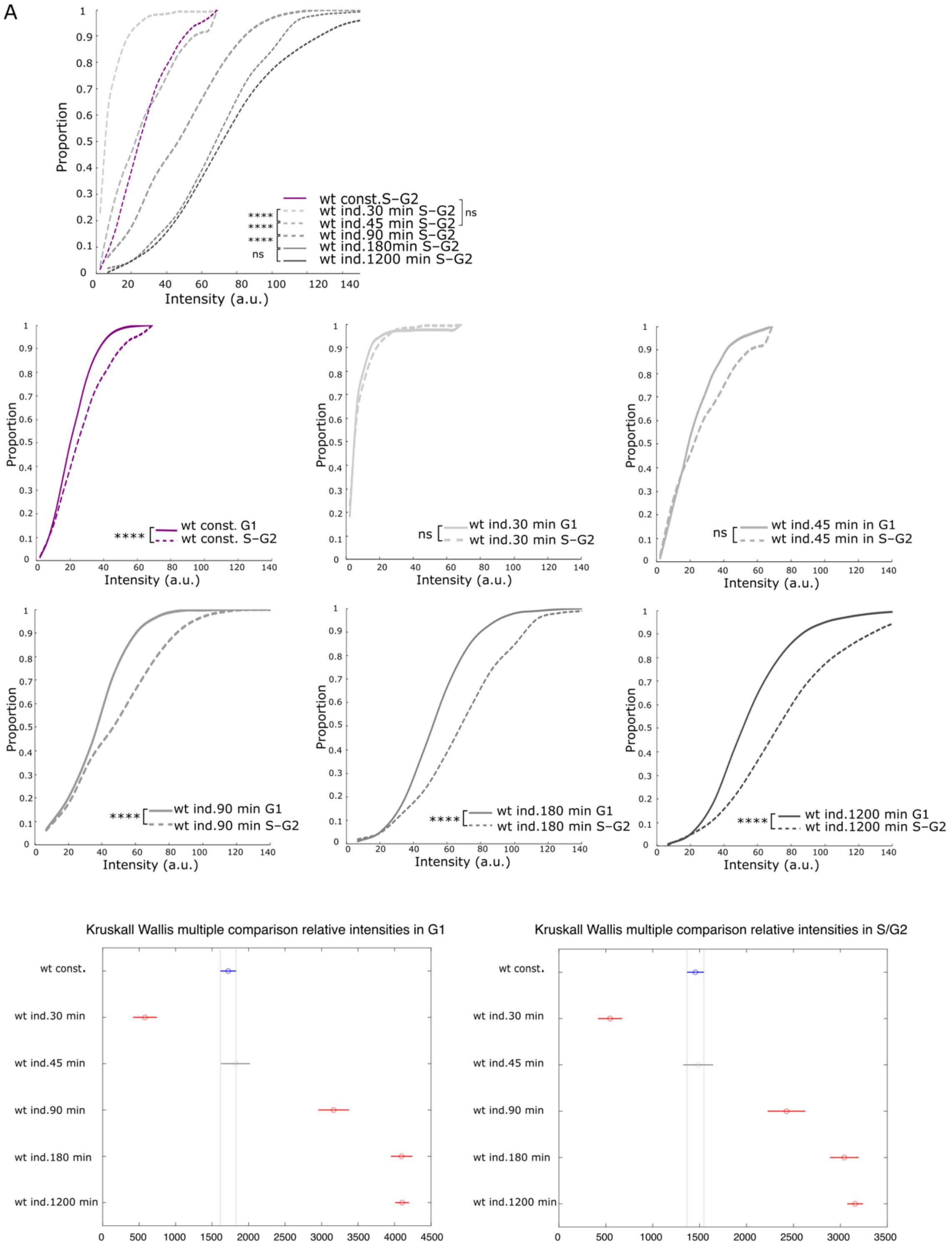

Related to Figure 2B

Related to Figure S2A

Groups with overlaping confidence intervals on the X axis are not significantly different at a significance level of 0.01

Loïodice et al. Figure S2 1/3

B

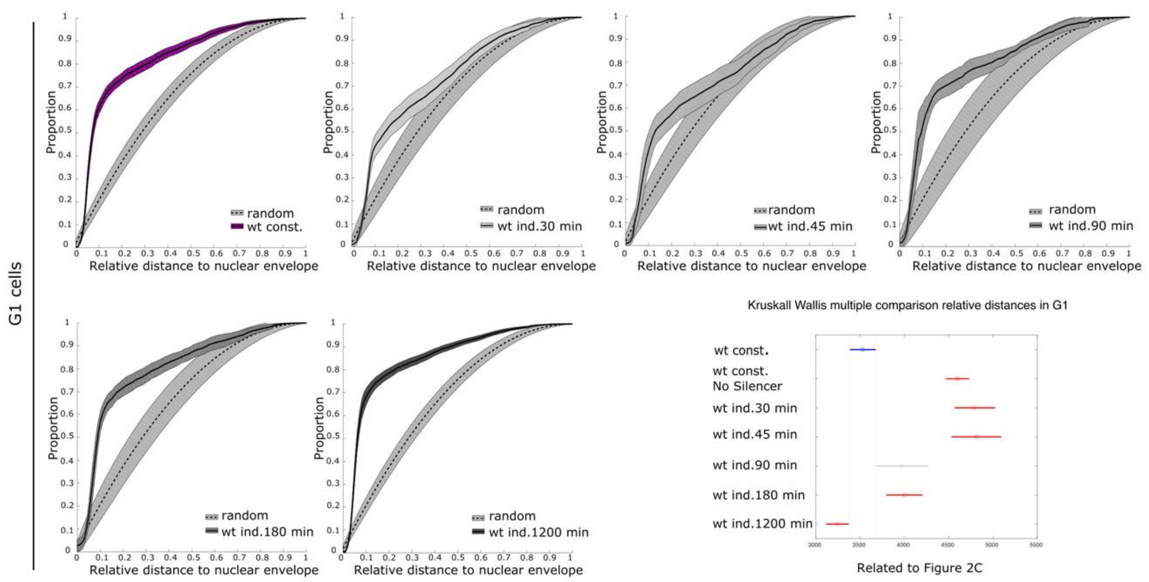

C

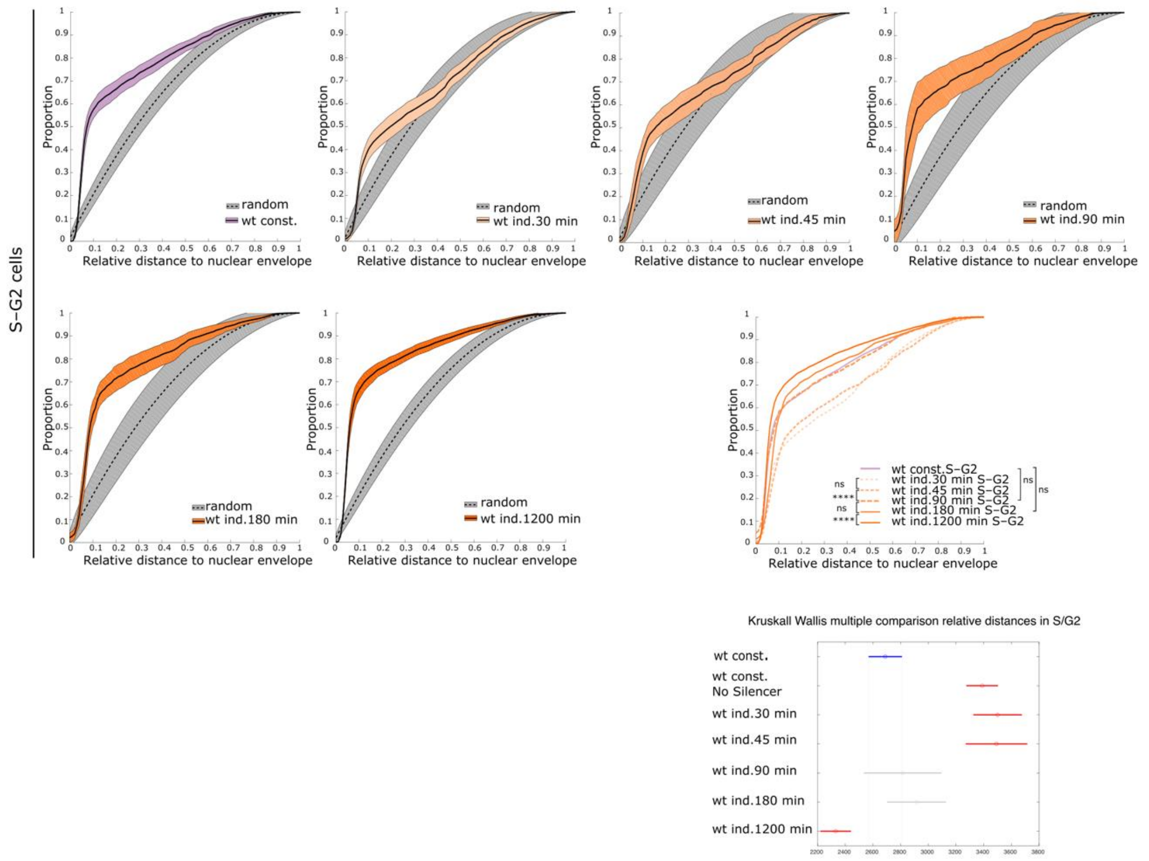

Loïodice et al. Figure S2 2/3

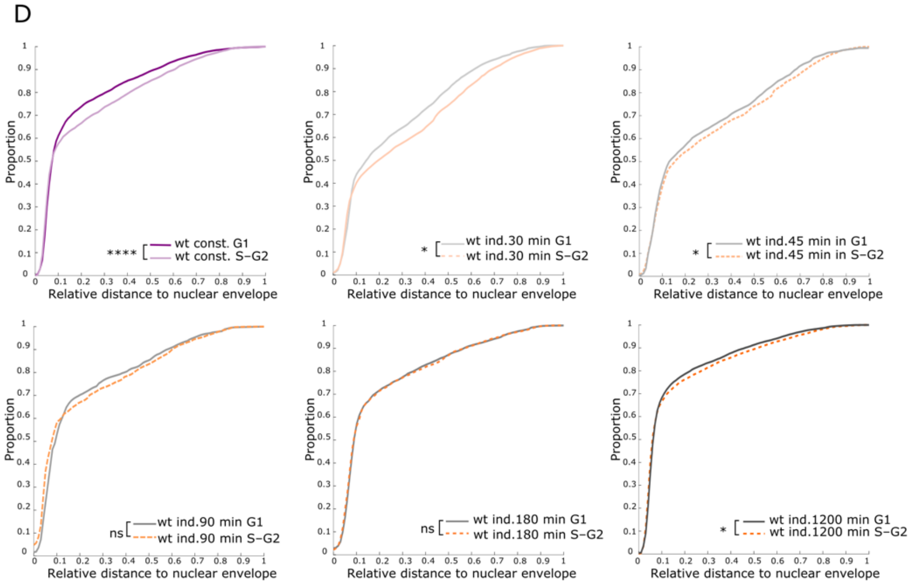

Loïodice et al. Figure S3/3

Figure S2 related to Figure 2. Distribution of intensities and localization of the *lys2::lacO E-ADE2-I* locus in G1 and S/G2 cells upon LacI induction. (**A**) Cumulative distributions of the intensity of GFP foci in S-G2 cells in strains bearing the *lys2::lacO E-ADE2-I* locus and expressing GFP-LacIR either under the constitutive *HIS3* promoter (wt const. yAT2059) or under the inducible *GALS* promoter (wt ind. yAT2078) at 30 min, 45 min, 90 min, 180 min, and 1200 min after galactose induction of the GFP-LacIR protein (upper panel). Cumulative distributions of the intensity of GFP foci in G1 and S-G2 for each time-point after galactose induction. And graphs of the estimates and comparison intervals obtained from Kruskal Wallis tests on the intensities of lacO/LacIR-GFP foci in G1 and in S-G2 cells for each condition (lower panels). p>0,01 is non significative (ns), p≤0,01 (*), p≤0,00001(****) and see Table S4 for statistics. (**B**) Cumulative distributions of the relative distance of *lys2::lacO E-ADE2-I* locus to the nuclear periphery in G1 cells, in strains expressing GFP-LacIR either under the constitutive *HIS3* promoter (wt const. yAT2059) grown in galactose medium or under the inducible *GALS* promoter (wt ind. yAT2078) at 30 min, 45 min, 90 min, 180 min, and 1200 min after galactose induction of the GFP-LacIR protein compared to a simulated random distribution for each condition. And graphs of the estimates and comparison intervals obtained from Kruskal Wallis tests on the relative distance of lacO/LacIR-GFP foci to the nuclear periphery in G1 cells for each condition. (**C**) Cumulative distributions of the relative distance of *lys2::lacO E-ADE2-I* locus to the nuclear periphery in S-G2 cells in strains expressing GFP-LacIR either under the constitutive *HIS3* promoter (wt const. yAT2059) grown in galactose medium or under the inducible *GALS* promoter (wt ind. yAT2078) at 30 min, 45 min, 90 min, 180 min, and 1200 min after galactose induction of the GFP-LacIR protein compared to a simulated random distribution for each condition. Cumulative distributions of the relative distance of *lys2::lacO E-ADE2-I* locus to the nuclear periphery in S-G2 cells. And graphs of the estimates and comparison intervals obtained from Kruskal Wallis tests on the relative distance of lacO/LacIR-GFP foci to the nuclear periphery in S-G2 cells for each condition p>0,01 is non significative (ns), p≤0,01 (*), p≤0,00001(****) and see Table S4 for statistics. (**D**) Cumulative distributions of the relative distance of *lys2::lacO E-ADE2-I* locus to the nuclear periphery, in G1 or S-G2 cells, in strains expressing GFP-LacIR either under the constitutive *HIS3* promoter (wt const. yAT2059) grown in galactose medium or under the inducible *GALS* promoter (wt ind. yAT2078) at 30 min, 45 min, 90 min, 180 min , and 1200 min after galactose induction of the GFP-LacIR protein for each condition. p>0,01 is non significative (ns), p≤0,01 (*), p≤0,00001(****) and see Table S4 for statistics.

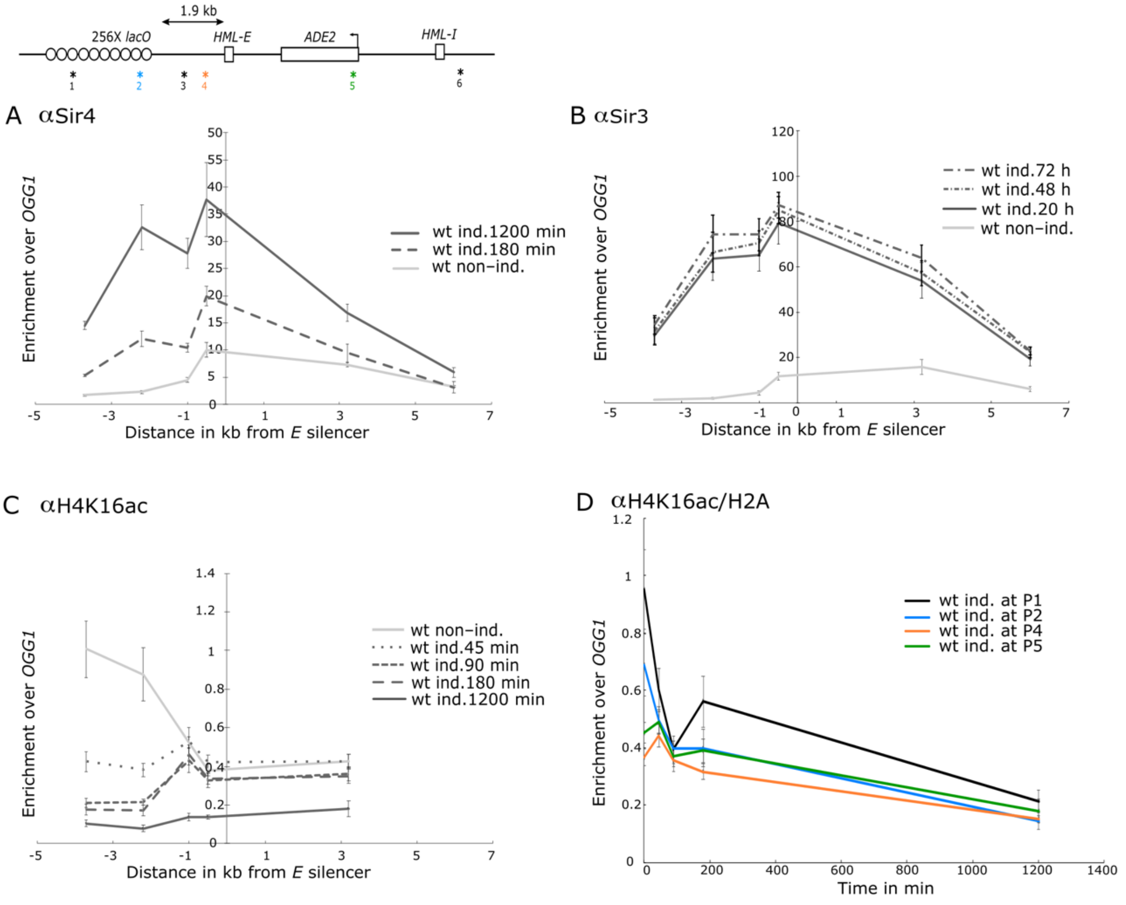

Loïodice et al. Figure S3

Figure S3 related to Figure 3. Heterochromatin formation takes place over several cell cycles and Sir3 recruitment plateaus after 24h. (**A**) Sir4 occupancy along the *lys2::lacO E-ADE2-I* locus, probed by ChIP-qPCR using an anti-Sir4 antibody developed by us (in this paper), in a strain expressing GFP-LacIR under the inducible *GALS* promoter (wt ind., yAT2078) in raffinose (no induction), or after 180 min, and 1200 min of galactose induction of the GFP-LacIR protein. (P1), (P2), (P3) (P4) amplicons are respectively located at 3.7 kb, 2.2 kb, 1 kb, and 0.5 kb from the left side of the *E* silencer, and (P5) and (P6) amplicons are located at 3.2 kb and 6 kb from the right side of the *E* silencer, amplicon positions are localized with an asterisk and their respective number on the scheme of the locus. The data were normalized over *OGG1* (shown as mean ± s.e.m. ; see Table S3 for the number of experiments). (**B**) Sir3 occupancy along the of *lys2::lacO E-ADE2-I* locus, probed by ChIP-qPCR using an anti-Sir3 antibody developed by us (Ruault et al., 2011), in strains expressing GFP-LacIR under the inducible *GALS* promoter (wt ind. yAT2078) in raffinose (no induction), or after 20 h, 48 h and 7 2h of galactose induction of the GFP-LacIR protein. The data were normalized over *OGG1* (shown as mean ± s.e.m.; see Table S3 for the number of experiments). (**C**) H4K16 acetylation occupancy along the of *lys2::lacO E-ADE2-I* locus, probed by ChIP-qPCR using an anti-acetyl-histone H4 (Lys16) antibody in a strain expressing GFP-LacIR under the inducible *GALS* promoter (wt ind., yAT2078) in raffinose (no induction), or after 45 min, 90 min, 180 min, and 1200 min of galactose induction of the GFP-LacIR protein. The data were normalized over *OGG1* (shown as mean ± s.e.m. ; see Table S3 for the number of experiments). (**D**) H4K16 acetylation enrichment over time at the (P1), (P2), (P4) and (P5) sites obtained by plotting data from Figure S3B when normalized by H2A ChIP signal. Error bars represent s.e.m (see Table S3 for the number of experiments).

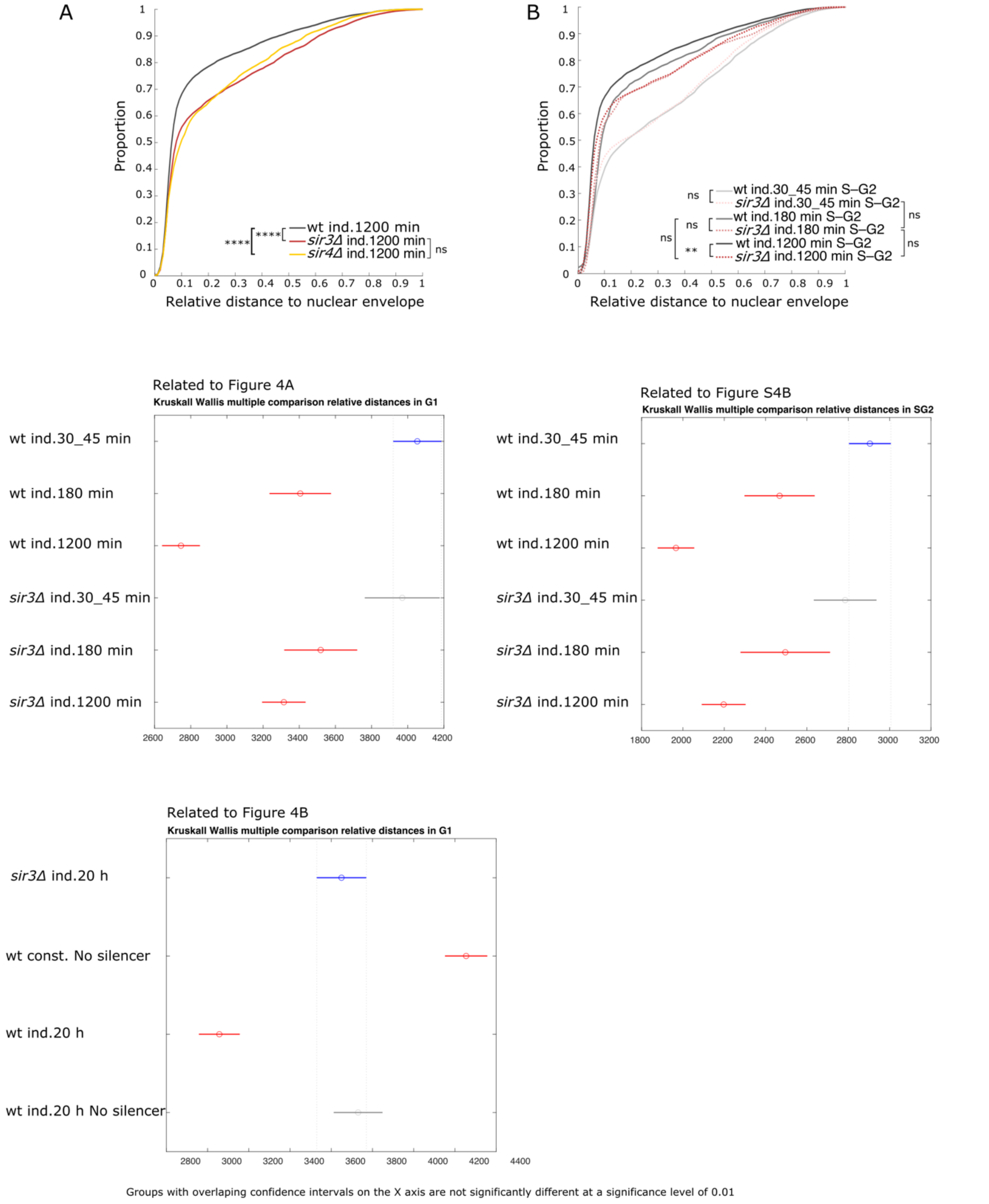

Loïodice et al. Figure S4

Figure S4 related to Figure 4. Perinuclear anchoring is partly Sir3 and Sir4 independent in G1 and S/G2 cells. (**A**) Cumulative distributions of the relative distance of *lys2:: lacO E-ADE2-I* locus to the nuclear periphery in G1 cells, in strains expressing GFP-LacIR under the inducible *GALS* promoter in the WT (wt ind., yAT2078), in a *sir3Δ* (*sir3Δ ind.*, yAT2370) and in a *sir4Δ* (*sir4Δ ind.*, yAT2370) after 1200 min of galactose induction. $p>0{,}01$ is non significative (ns), $p\leq 0{,}00001$(****) and see Table S4 for statistics. (**B**)

Cumulative distributions of the relative distance of *lys2:: lacO E-ADE2-I* locus to the nuclear periphery in S-G2 cells, in strains expressing GFP-LacIR under the inducible *GALS* promoter in the WT (wt ind., yAT2078) and in a *sir3Δ* (*sir3Δ ind.,* yAT2370) after 30_45 min, 180 min, and 1200 min of galactose induction. p>0,01 is non significative (ns), p≤0,001 (**), and see Table S4 for statistics. And graphs of the estimates and comparison intervals obtained from Kruskal Wallis tests on the relative distance of lacO/LacI-GFP spots to the nuclear periphery in G1 and in S-G2 cells for each condition (lowel panels).

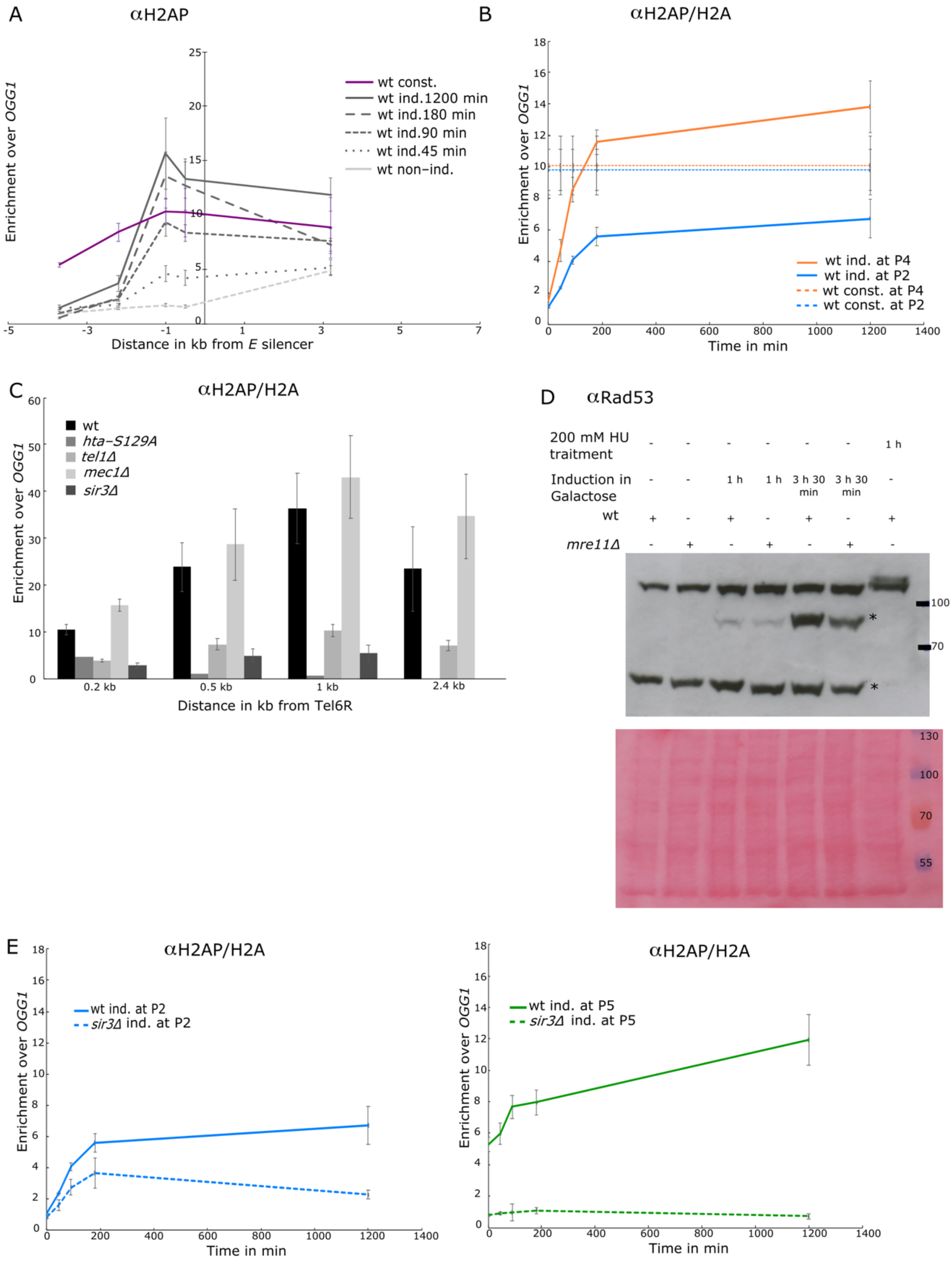

Loïodice et al. Figure S5 1/2

F

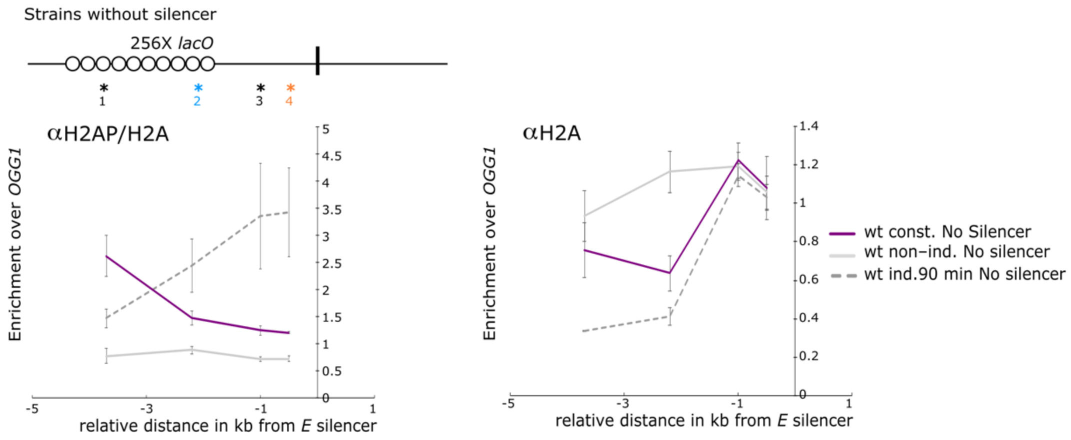

G

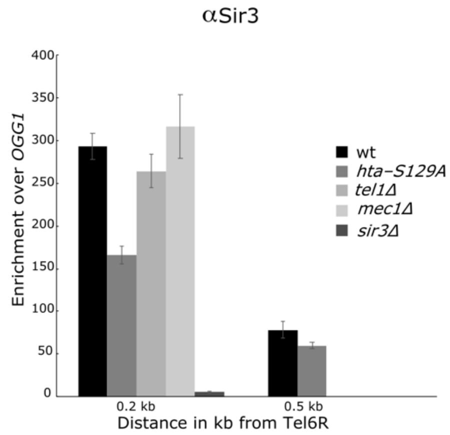

H

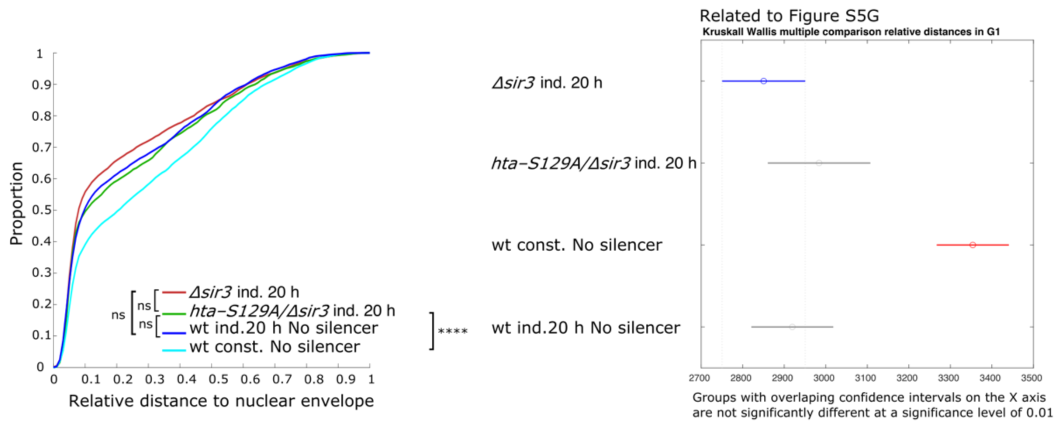

Loïodice et al. Figure S5 2/2

Figure S5 related to Figure 5. LacI binding induces a transient H2A phosphorylation without detectable Rad53 phosphorylation. (**A**) H2A-S129 phosphorylation occupancy along the *lys2::lacO E-ADE2-I* locus probed by ChIP-qPCR using an anti-H2A Phospho-S129 antibody in strains expressing either the GFP-LacIR under the constitutive *HIS3* promoter (wt const., yAT2059) or under the *GALS* promoter (wt ind.,

yAT2078) in raffinose (no induction), or after 45 min, 90 min, 180 min, and 1200 min of galactose induction. The data were normalized over *OGG1* (shown as mean ± s.e.m. ; see Table S3 for the number of experiments). (**B**) H2A-S129 phosphorylation enrichment over time at (P2) and (P4) obtained by plotting the data from Figure 5A. (**C**) H2A-S129 phosphorylation occupancy at 0,2 kb, 0,5 kb, 1 kb and 2,4 kb from the end of telomere 6R, in strains bearing the *lys2::lacO E-ADE2-I* locus, probed by ChIP-qPCR using an anti-H2A Phospho-S129 antibody and expressing GFP-LacIR under the inducible *GALS* promoter in the WT (wt ind., yAT2078), in a *hta-S129A* mutant (*hta-S129A* ind, yAT2965) as a control for the background of the ChIP experiment, in a *tel1Δ* (*tel1Δ ind,.* yAT2314), in a *mec1Δ* (*mec1Δ ind.*, yAT2904), and in a *sir3Δ* (*sir3Δ ind,.* yAT2370) strains. The data were normalized over *OGG1* and histone H2A ChIP signal (shown as mean ± s.e.m. ; see Table S3 for the number of experiments). (**D**) Immunoblots with anti-Rad53 antibody (Rad53 (yC-19):sc-6749, Santa Cruz) on crude extracts from strains expressing GFP-LacIR under the inducible *GALS* promoter in a WT (wt ind. No Silencer, yAT1909) and *mre11Δ* (*mre11Δ* ind. No Silencer, yAT2142) strains, in raffinose (no induction) or after 1 h and 3 h 30 min of galactose induction. As a positive control of full activation of Rad53, WT strain (yAT1798) expressing GFP-LacI under *HIS3* promoter was treated with 200 mM HU (hydroxyurea, Sigma-Aldrich). * Interpretated as unspecific bands related to growth media. (**E**) H2A-S129 phosphorylation enrichment over time at the *lacO* (P2) (left panel) and in the body of *ADE2* gene (P5) (right panel) in strains bearing the *lys2::lacO E-ADE2-I* locus, probed by ChIP-qPCR using an anti-H2A Phospho-S129 antibody in strains expressing GFP-LacIR under the *GALS* promoter in the WT (wt ind., yAT2078), and in a *sir3Δ* (*sir3Δ ind,.* yAT2370) strains in raffinose (no induction), or after 45 min, 90 min, 180 min, and 1200 min of galactose induction. The data were normalized over *OGG1* and histone H2A ChIP signal (shown as mean ± s.e.m.; see Table S3 for the number of experiments). (**F**) H2A-S129 phosphorylation (upper panel) and H2A (lower panel) occupancy along the *lys2::lacO array* locus (No Silencer) probed by ChIP-qPCR using anti-H2A Phospho-S129 and anti-H2A antibodies in strains expressing either GFP-LacIR under the constitutive *HIS3* promoter (wt const. No silencer, yAT1798) or under the control of the inducible *GALS* promoter (wt ind. No Silencer, yAT1909) in raffinose (no induction), or after 90 min of galactose induction. The data were normalized over *OGG1* and H2A-S129 phosphorylation was also normalized over histone H2A ChIP signal (shown as mean ± s.e.m.; see Table S3 for the number of experiments). (P1), (P2), (P3) and (P4) amplicons are respectively located at 3.7 kb, 2.2 kb, 1 kb and 0.5 kb from the left side of the theoretical location of the E silencer. (**G**) Sir3 occupancy at 0,2 kb, and 0,5 kb from the end of telomere 6R in strains bearing the *lys2::lacO E-ADE2-I* locus, probed by ChIP-qPCR using anti-Sir3 antibody, and expressing GFP-LacIR under the control of the *GALS* promoter in the WT (wt ind., yAT2078), in a *hta-S129A* (*hta-S129A* ind, yAT2965), and Sir3 occupancy at 0,2 kb from the end of telomere 6R in a *tel1Δ* (*tel1Δ ind,.* yAT2314), in a *mec1Δ* (*mec1Δ ind.*, yAT2904), and in a *sir3Δ* (*sir3Δ ind,.* yAT2370) strains. The data were normalized over *OGG1* (shown as mean ± s.e.m. ; see Table S3 for the number of experiments). (**H**) Left panel: Cumulative distributions of the relative distance of *lys2::lacO E-ADE2-I* locus to the nuclear periphery in G1 cells, in strains expressing GFP-LacIR under the inducible *GALS* promoter in a *sir3Δ* (*Δsir3 ind.*, yAT2370) and in a double mutant *hta-S129A/ sir3Δ* (*hta-S129A/ sir3Δ* ind, yAT2995) strains after 20 h of galactose induction; and cumulative distributions of the relative distance of the *lys2::lacO* array locus (No Silencer) locus to the nuclear periphery in G1 cells, in strains expressing GFP-LacIR either under the inducible *GALS* promoter (wt ind. No Silencer, yAT3471) after 20 h of galactose induction or under the constitutive *HIS3* promoter (wt const. No Silencer, yAT3420). p>0,01 is non significative (ns), p≤0,00001(****) and see Table S4 for statistics. Right panel: graphs of the estimates and comparison intervals obtained from Kruskal Wallis tests on the relative distance of lacO/LacI-GFP spots to the nuclear periphery in G1 cells for each condition (lower panel).

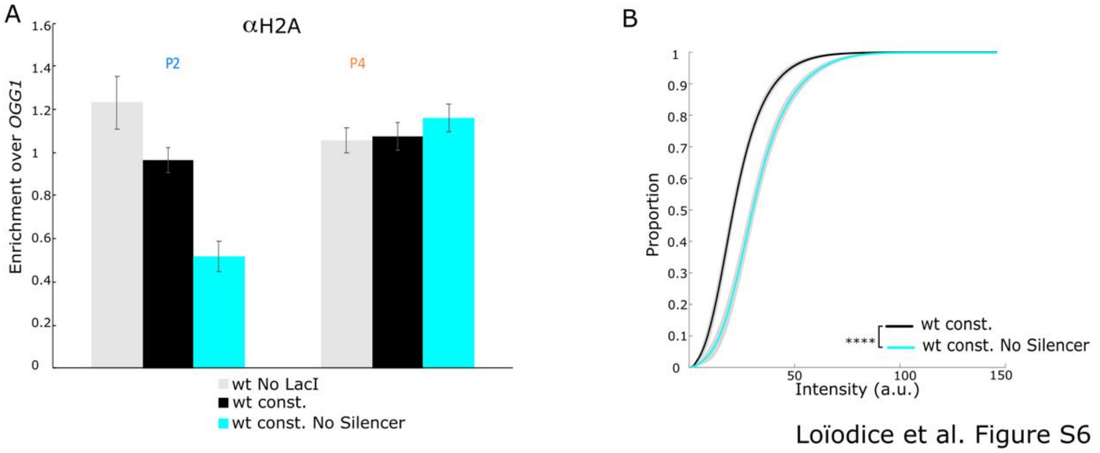

Figure S6 related to Figure 6. SIR spreading stabilizes nucleosome and limits LacI access to DNA. (**A**) H2A occupancy at the lacO (P2) and nearby the *E* silencer (P4), probed by ChIP-qPCR using an anti-H2A antibody, in strains bearing the *lys2::lacO E-ADE2-I* locus either without LacI expression (wt No LacI, yAT2001) or expressing GFP-LacIR under the constitutive *HIS3* promoter (wt const., yAT2059) strain; and in a strain bearing the *lys2::lacO* array locus (No silencer) and expressing GFP-LacIR under the constitutive *HIS3* promoter (wt. const No Silencer yAT3420). The data were normalized over *OGG1* (shown as mean ± s.e.m. ; see Table S3 for the number of experiments). (**B**) Cumulative distributions of the intensity of GFP foci in G1 cells, expressing GFP-LacIR under the constitutive *HIS3* promoter in strains bearing either the *lys2::lacO E-ADE2-I* locus WT strain (wt const. yAT2059) or the *lys2::lacO* array locus (wt const. No silencer, yAT3420). p>0,01 is non significative (ns), p≤0,00001(****) and see Table S4 for statistics.

References

1. Thomas, B.J.; Rothstein, R. Elevated Recombination Rates in Transcriptionally Active DNA. *Cell* **1989**, *56*, 619–630, doi:10.1016/0092-8674(89)90584-9.
2. Ruault, M.; De Meyer, A.; Loïodice. I.; Taddei, A. Clustering heterochromatin: Sir3 promotes telomere clustering independently of silencing in yeast. *J Cell Biol*. **2011** *192*, 417-431. doi: 10.1083/jcb.201008007.
3. Masumoto, H.; Hawke, D.; Kobayashi, R.; Verreault, A. A role for cell-cycle-regulated histone H3 lysine 56 acetylation in the DNA damage response. *Nature*. **2005**, *436*, 294-2988. doi: 10.1038/nature03714.
4. Winston, F.; Dollard, C.; Ricupero-Hovasse, S.L. Construction of a Set of Convenient *Saccharomyces cerevisiae* Strains That Are Isogenic to S288C. *Yeast* **1995**, *11*, 53–55, doi:10.1002/yea.320110107.
